# Fatty acid amide hydrolase in major depressive episodes: A [^11^C]CURB positron emission tomography study

**DOI:** 10.1038/s41386-025-02150-y

**Published:** 2025-06-23

**Authors:** Dorsa Rafiei, Michelle De Pol, Jeffrey H. Meyer, Kimberly Desmond, Ruth Ross, Isabelle Boileau, Jerry Warsh, Pablo Rusjan, Neil Vasdev, Ryan Aloysius, Lauren Gray, Nathan J. Kolla

**Affiliations:** 1 Brain Health Imaging Centre, Campbell Family Mental Health Research Institute, The Centre for Addiction and Mental Health, Toronto, ON Canada; 2 Azrieli Centre for Neuro-Radiochemistry, Campbell Family Mental Health Research Institute, The Centre for Addiction and Mental Health, Toronto, ON Canada; 3 Department of Pharmacology and Toxicology, University of Toronto, Toronto, ON Canada; 4 Department of Psychiatry, University of Toronto, Toronto, ON Canada; 5 Douglas Research Centre, McGill University, Montreal, QC Canada; 6 Department of Psychiatry, McGill University, Montreal, QC Canada; 7 Swinburne University of Technology, Centre for Forensic Behavioural Science, Melbourne, VIC Australia

**Keywords:** Depression, Predictive markers

## Abstract

The role of the endocannabinoid system (ECS) in major depressive disorder (MDD) is under-investigated despite reports of increased activity and/or concentration of fatty acid amide hydrolase (FAAH), a key ECS enzyme, in fronto-limbic brain regions in some animal models of depressive behavior. We hypothesized that [^11^C]CURB λ*k*_3_, an index of FAAH density, would be elevated in the prefrontal cortex, hippocampus, and anterior cingulate cortex in major depressive episodes of MDD compared to healthy controls. Fifteen unmedicated MDD participants and 15 age- and sex-matched healthy controls underwent [^11^C]CURB positron emission tomography and FAAH genotyping. Psychological tests of depressive severity, apathy, and anxiety were administered and measurements were assessed as covariates in exploratory analyses. No significant group differences in [^11^C]CURB λ*k*_3_ were observed between MDD participants and controls (*F*_1,27_ = 0.32; *p* = 0.58). A mixed effects model revealed that Marin Apathy Evaluation Scale scores in the MDD group had a significant main effect on [^11^C]CURB λ*k*_3_ binding across the collective regions of medial prefrontal cortex, orbitofrontal cortex, anterior cingulate cortex, ventral striatum, and midbrain (*F*_1,11_ = 6.75; *p* = 0.02). Depressive severity and anxiety did not have a significant relationship to [^11^C]CURB λ*k*_3_ binding. The relationship of greater fronto-limbic [^11^C]CURB λ*k*_3_ to greater apathy along with the metabolic role of FAAH in the ECS, the latter which supports maintaining feelings of interest, initiative, and motivation, has important implications for the pathophysiology of apathy in MDD.

## Introduction

Major depressive disorder (MDD) affects approximately 280 million people and is one of the leading causes of disability worldwide [1, 2]. Fifty percent of cases are treatment-resistant to first- and second-line monoamine-targeting antidepressants, such as selective serotonin reuptake inhibitors and serotonin-norepinephrine reuptake inhibitors; and while esketamine and ketamine help some patients with MDD, approximately 30% of cases do not respond to these medications [3, 4]. A plausible explanation for treatment resistance is that MDD is heterogeneous and that there are as yet undetermined pathologies that remain untargeted by current pharmacological approaches. Hence, identifying such pathologies and eventually developing novel, targeted therapeutics may address these shortcomings.

Accumulating evidence suggests that dysregulation of the endocannabinoid system (ECS), which has functional roles in mood, motivation, appetite control, and memory processing, may contribute to depressive symptoms [5, 6]. Fatty acid amide hydrolase (FAAH) is an ECS enzyme that metabolizes several fatty acid amides; and is the principal metabolic enzyme of N-arachidonoylethanolamine (AEA, or anandamide), which is an endogenous ligand of cannabinoid 1 (CB_1_) receptors [7]. In some preclinical models of depressive behavior, FAAH activity, mRNA, and protein are increased in regions that participate in affect and motivation, such as the prefrontal cortex (PFC), hippocampus, and striatum [8, 9]. In humans, the CB_1_ receptor antagonist, rimonabant, is associated with an increased risk of developing or exacerbating depressive symptoms [10]. Post-mortem studies of MDD patients, albeit still in a preliminary phase of investigation, report increased neural CB_1_ receptor mRNA and protein in the PFC, consistent with a model of elevated FAAH activity, reduced AEA; and less stimulation of CB1 receptors with their upregulation [11, 12]. There is also considerable interest in the therapeutics of FAAH inhibition. For example, AEA-CB_1_ signaling has been associated with anti-depressive and anxiolytic-like effects as well as stress in rodents [13–15]. In response to stressors, such as in the chronic unpredictable stress [16] or the chronic mild stress models of depression [8], FAAH can initiate the degradation of AEA, thus reducing AEA-CB_1_ signaling. Furthermore, genetic knockout or pharmacological inhibition of FAAH was found to reduce depressive-like behavior in rodents [17, 18]. Presently, a key gap in the literature is an absence of investigations of brain FAAH in major depressive episodes (MDE) of MDD.

Positron emission tomography (PET) imaging with [^11^C-carbonyl]6-hydroxy-[1,1′-biphenyl]-3-yl cyclohexylcarbamate ([^11^C]CURB), is the most advanced method to detect λ*k*_3_, an index of brain FAAH levels in vivo [19]. [^11^C]CURB has high selectivity and high brain uptake; and radiolabelled metabolites do not cross the blood brain barrier [20]. [^11^C]CURB is modelled with an irreversible two-tissue compartment kinetic model; and there is no effect of blood flow on λ*k*_3_ [21]. FAAH levels are associated with FAAH activity, as demonstrated by paradigms that influence FAAH levels, such as the rs324420 variant and models of under- or overexpression of the *FAAH* gene [22–24].

Several preclinical models of depressive behavior have reported elevated FAAH within fronto-limbic brain regions, and *FAAH* gene over-expression has led to depressive-like behavior and a reduction in AEA [25, 26]. Evidence of FAAH elevation in fronto-limbic regions may translate to humans with MDD; thus, the main objective of this study was to compare [^11^C]CURB λ*k*_3_ in the PFC, anterior cingulate cortex (ACC), and hippocampus in MDD with a current MDE versus healthy controls. Our primary hypothesis is that [^11^C]CURB λ*k*_3_ will be elevated in the MDD group relative to controls. These regions were chosen as our primary brain regions of interest (ROI) because these regions are known to have a high density of FAAH [27] and also participate in emotion and affect regulation [28, 29].

λ*k*_3_ is the measured index of FAAH density. It is derived from a two-compartment modeling solution in brain tissue of [^11^C]CURB transfer from arterial plasma in which one compartment represents free and non-specific binding and the other specific binding in which there is negligible transfer from specific binding to free and non-displaceable binding compartments. *k*_3_ is the imaging agent transfer from the free and non-specifically bound compartment to the specific binding compartment and λ is the ratio of free and non-specifically bound imaging agent to arterial blood at equilibrium. λ*k*_3_ is also a desirable measure because it is not related to blood flow, previously demonstrated with arterial spin labelling a property attributable to a *k*_3_/*k*_2_ ratio of approximately 0.55 in contrast to most other irreversible PET imaging agents in which *k*_3_>>*k*_2_ [21].

Given that the endocannabinoid system is known to influence response to stress, mood regulation, level of anxiety and motivation in animals involving a substantial number of behaviors [15, 25, 26, 30–32], in our study design we prioritized behaviors that had greater demonstration in humans, were more specifically related to alterations in available FAAH level and/or more present in MDD. Notable examples within this scope include reports that CB1 antagonism may induce depressive symptoms [10], FAAH inhibitor JNJ-42165279 had anxiolytic effects in social anxiety disorder [33] and cannabis use may be associated with apathy [34]. Another set of notable findings is that loss of FAAH from destabilization related to a single nucleotide polymorphism (rs324420) in humans is associated with reduced anxiety [35], and increased behaviors fostered by greater motivation [36]. Apathy and anxiety are present in the majority of MDEs [37, 38].

We note that apathy has been succinctly defined as a lack of motivation, but more recent descriptions include a sustained quantitative reduction of goal directed activity accompanied by diminished emotional reactions and/or social engagement [39, 40]. Apathy is important in MDD as it is present in the majority of cases [38, 41] but it is not used as diagnostic criterion due to lack of clinical specificity [42]. The Marin Apathy Evaluation Scale is a commonly applied scale to assess apathy with excellent internal consistency, reliability, and is elevated in populations reporting apathy such as those with MDEs [38]. Anhedonia is part of the definition of an MDE and relates to apathy since it is a loss of interest or pleasure in previously rewarding activities [43].

Considering the above evidence and the functional circuits involved in depression, anxiety, and apathy, there are several exploratory hypotheses in the present study. First, we hypothesized that depressive severity has a positive association with [^11^C]CURB λ*k*_3_ in brain regions of the MDD group implicated in regulating depressed mood, including the PFC, ACC, and hippocampus. Second, in the MDD group, we hypothesized that anxiety is positively associated with [^11^C]CURB λ*k*_3_ in brain regions related to such, including the orbitofrontal cortex (OFC), ACC, hippocampus, insula, and amygdala. Third, in the MDD group, we anticipated that apathy would be positively associated with [^11^C]CURB λ*k*_3_ in brain regions whose altered function is implicated in apathy, including the medial prefrontal cortex (mPFC), OFC, ACC, ventral striatum, and midbrain (including the substantia nigra and ventral tegmental area).

## Materials and methods

All participants provided written informed consent before initiating the study procedures. All components of the study were approved by Health Canada and the Research Ethics Board of the Centre for Addiction and Mental Health (CAMH) in Toronto, Ontario, Canada.

### Participants

Fifteen MDD participants with a current MDE and 15 age- and sex-matched healthy controls completed the study protocol. All participants were recruited from the local community in Toronto, Canada between January 2021 and July 2023. All psychiatric diagnoses were verified with the Structured Clinical Interview for DSM-5 – Research Version (SCID-5-RV) by trained raters [44], and then reviewed and confirmed by a psychiatrist (NJK). Individuals with MDD were included if they scored greater than or equal to 17 on the 17-item Hamilton Depression Rating Scale (HDRS) [45]. Exclusion criteria for the MDD participants included use of psychotropic medication in the past six weeks; history of bipolar or schizophrenia spectrum disorder; antisocial or borderline personality disorder; and history of alcohol or substance abuse in the past 12 months, as confirmed by the SCID-5-RV [44]. Healthy controls were matched to MDD participants by age within 5 years, sex, and rs324420 genotype [23], the latter which influences [^11^C]CURB λ*k*_3_ (see FAAH Genotyping later in methods section).

Exclusion criteria for healthy controls included a score greater than seven (which represents the threshold for health) on the HDRS or any lifetime history of psychiatric disorders or psychotropic medication use. Neurological illness, head trauma, a positive drug screen for drugs of abuse, pregnancy in females, and contraindications to safe magnetic resonance imaging (MRI) scanning also precluded participation in both MDD and healthy cohorts.

Depressive severity, apathy, and anxiety were assessed with the 17-item HDRS [45], the Marin Apathy Evaluation Scale Self Version (MAES) [38], and the Hamilton Anxiety Rating Scale (HAM-A) respectively [46].

### Image acquisition and analysis

The radiosynthesis of [^11^C]CURB was completed as previously described by our laboratories [19]. Participants wore a thermoplastic mask to reduce head movement for the duration of the PET scan. Each participant completed one [^11^C]CURB PET scan in a Discovery MI PET-CT scanner (General Electric, Milwaukee, WI, USA) following an intravenous injection of 370 ± 40 MBq (10 ± 1 mCi) of [^11^C]CURB [47]. Brain radioactivity was computed during sequential frames of increasing duration, and the scan time was 60 min to allow sufficient time for λ*k*_3_ to stabilize [21]. Due to the widespread distribution of FAAH in the brain, there is no adequately large reference region to quantify [^11^C]CURB, necessitating the measurement of the arterial input function [21, 26]. Following the [^11^C]CURB injection, arterial blood was sampled continuously for the first 22.5 min with an automatic blood sampling system (ABSS Model PBS-101, Veenstra Instruments, Joure, Netherlands) and manual samples of 4–10 mL were also collected at 3, 7, 12, 20, 30, 45, and 60 min. Radioactivity of whole blood and plasma aliquots from the manual and automatic samples were measured in a gamma counter. The whole blood-to-plasma radioactivity ratio, interpolated by a biexponential function and parent plasma fraction fit with a Hill function, was applied to create the metabolite-corrected arterial input function [21].

In addition, each participant completed a standard T1-weighted brain MRI scan (TR = 6.7 ms, TE = 3 ms, TI = 650 ms, flip angle=8°, field of view = 230 × 230 mm, matrix size = 256 × 256 × 200, voxel size=0.9 mm), acquired on a Discovery MR750 3 T MRI scanner (General Electric, Milwaukee, WI, USA) for ROI delineation. The ROIs were transformed onto the PET image from the co-registration parameters of the T1 MRI to the motion-corrected PET image (PMOD Technologies LLC, RRID: SCR_016547, version 4.202). Time activity curves (TACs) were extracted from each ROI, and an irreversible two-tissue compartment model was used, along with the arterial input function, to fit the TACs, generating the composite rate constant λ*k*_3_ (λ = *K*_1_/*k*_2_) to quantify [^11^C]CURB binding as previously validated [21].

### *FAAH* polymorphism genotyping

Baseline FAAH protein levels are known to be affected by a single nucleotide polymorphism in the human *FAAH* gene (rs324420) that substitutes a cytosine for an adenine nucleoside (C385A), which results in the substitution of a proline to a threonine at amino acid position 129 (P129T). Relative to the C/C genotype, those homozygous or heterozygous for the A allele have similarly reduced [^11^C]CURB binding (λ*k*_3_) in the brain [23]. The *FAAH* rs324420 variant was genotyped for all participants according to the manufacturer’s directions for a TaqMan SNP Genotyping assay (ID C_1897306_10; Life Technologies, Burlington, ON, Canada) on a ViiA7 instrument (Life Technologies, Burlington, ON, Canada) using 20 ng total genomic DNA template, Perfecta FastMix II (Quantabio, Beverly, MA, USA), in a total reaction volume of 10 µL.

### Statistical analysis

To analyze [^11^C]CURB λ*k*_3_ between groups, a linear mixed effects model was used with diagnosis and genotype included as fixed factors, regional [^11^C]CURB λ*k*_3_ as the repeated within-subjects measure, and participant as a random effect. The prioritized group comparison included the subregions of the PFC, ACC, and hippocampus. A secondary linear mixed effects model compared groups across regional [^11^C]CURB λ*k*_3_ in a broader range of grey matter regions throughout the brain, including the amygdala, ventral striatum, putamen, dorsal caudate, temporal cortex, occipital cortex, insula, thalamus, substantia nigra, pons, and cerebellum. To assess our exploratory hypotheses, three linear mixed effects models were applied to the MDD sample, with either HDRS, MAES, or HAM-A total scores as a covariate, genotype as a fixed factor, brain region as a repeated measure, and participant as a random effect. For the primary outcome measure, a threshold of *p* = 0.05 was applied for significance. Our three exploratory outcomes were effect of HDRS, effect of MAES, and effect of HAM-A on λ*k*_3_ in a group of regions creating three additional analyses. However, none of these analyses are independent: Effect of diagnosis is related to effect of HDRS as diagnosis corresponds to a threshold of HDRS. Both the MAES and HAM-A can be correlated with total severity of HDRS in large samples. So, the latter measures, reflecting approximately two analyses were given a meaningful significance threshold of *p* = 0.025. In addition, Partial Pearson correlation coefficients were utilized to analyze [^11^C]CURB λ*k*_3_ and symptom measures in each individual brain ROI, controlling for genotype.

## Results

### Demographics

Fifteen MDD participants and 15 healthy controls were included in the study (Table 1). There were 11 females (73%) and 4 males (27%) in each group. The mean age was 26.6 years (range: 19 to 47 years) for MDD participants and 25.3 years (range: 19 to 43 years) for healthy controls. There were no significant differences between groups regarding race, body mass index, or years of education. Ten participants in each group (67%) carried the C/C genotype and five participants in each group (33%) carried the heterozygous A/C genotype. All participants were free from cannabis use for at least four weeks prior to study enrolment as verified by self-report and urine drug screen. There were no group differences in radiotracer injected dose (7.36 ± 6.39 vs. 8.92 ± 9.33 nmol; *t* = -0.53; *p* = 0.60) and specific activity at the time of injection (74.5 ± 42.4 vs. 75.0 ± 45.4 TBq/mmol; *t* = -0.03, *p* = 0.98) (Table S1).

**Table 1:** Clinical and demographic variables.

	MDD (*n* = 15)	HC (*n* = 15)	Statistics	*P* value
**Age** (years)^a^	26.6 ± 7.80	25.3 ± 6.64	*t* = 0.50	0.61
**Sex** (Female:Male)^b^	11:04	11:04	—	1
**Education** (years)^a^	15.3 ± 1.62	15.9 ± 1.87	*t* = −1.04	0.98
**Race**^c^			*X*^*2*^ = 1.35	0.85
Caucasian (*n*)	4	3	—	—
Black (*n*)	0	1	—	—
East Asian (*n*)	6	4	—	—
South Asian (*n*)	4	6	—	—
Middle Eastern (*n*)	1	1	—	—
**Body Mass Index** (kg/m^2^)^a^	24.3 ± 4.53	25.0 ± 5.04	*t* = −0.42	0.68
***FAAH*** **Genotype**	10:05:00	10:05:00	—	—
(C/C:A/C:A/A)				
**Number of DSM-5 MDE Symptoms**	7.8 ± 1.01	—	—	—
**Duration of Current MDE** (months)	9.0 ± 8.00	—	—	—
**Number of Past MDEs**	7.1 ± 9.55	—	—	—
**Age of Onset of MDD** (years)	15.7 ± 4.27	—	—	—
**Hamilton Depression Rating Scale**^d^	20.5 ± 4.64	—	—	—
**Hamilton Anxiety Rating Scale**	17.1 ± 5.59	—	—	—
**Marin Apathy Evaluation Scale**	41.0 ± 6.58	—	—	—

Data presented as mean ± standard deviation, unless stated otherwise.

^a^Independent Samples *t*-test.

^b^Fisher’s Exact Test.

^c^Chi-Square Test.

^d^17-item Hamilton Depression Rating Scale.

### Comparison of [^11^C]CURB λ*k*_3_ between MDD and healthy

**Fig. 1:**
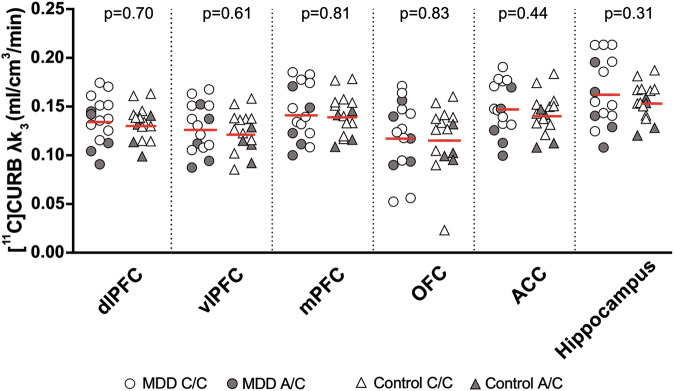
[^11^C]CURB λ*k*_3_ across prioritized brain regions in MDD and healthy control groups. There was no significant group effect of [^11^C]CURB λ*k*_3_ between MDD participants and healthy controls (*F*_1,27_ = 0.32, *p* = 0.58). Red bars indicate means, adjusted for genotype. dlPFC dorsolateral prefrontal cortex, vlPFC ventrolateral prefrontal cortex, mPFC medial prefrontal cortex, OFC orbitofrontal cortex, ACC anterior cingulate cortex.

Contrary to our primary hypothesis, a linear mixed effects model demonstrated no significant group effect of [^11^C]CURB λ*k*_3_ between MDD participants and healthy controls in the repeated measures of PFC, ACC, and hippocampus (*F*_1,27_ = 0.32, *p* = 0.58; Fig. 1). There was no significant effect of sex on [^11^C]CURB λ*k*_3_ (*F*_1,27_ = 0.17, *p* = 0.69). As an additional analysis we applied the same linear mixed effects model and found no significant group effect of [^11^C]CURB λ*k*_3_ between MDD participants and healthy controls across all brain ROIs (*F*_1,27_ = 0.33, *p* = 0.57; Table S2). An additional, exploratory, linear mixed effects model evaluating effect of rs324420 genotype across the sample found that C/C carriers had significantly higher λ*k*_3_ values in the primary regions of interest as compared to A/C (F_1,28_ = 5.97, p = 0.02).

### Relationship of psychological measures to [^11^C]CURB *λk*_3_

**Fig. 2:**
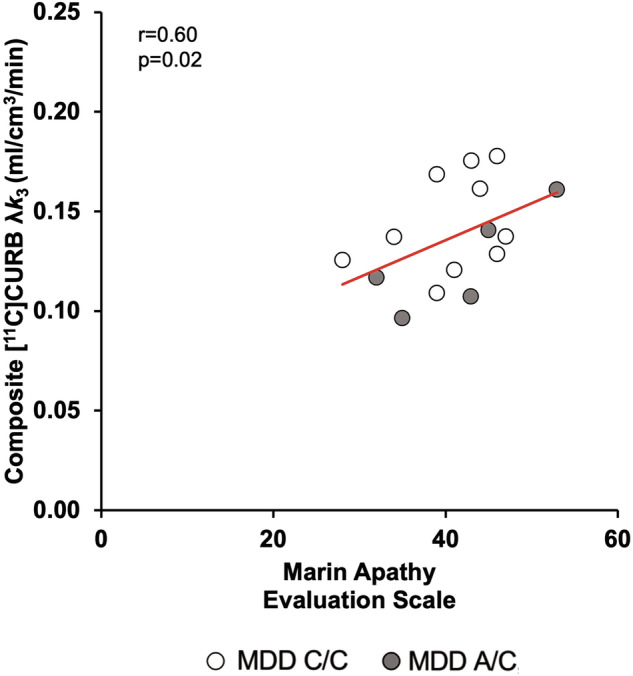
Positive correlation between Marin Apathy Evaluation Scale (MAES) scores and a composite region of [^11^C]CURB λ*k*_3_ in the MDD group. MDD participants who had higher MAES scores also had greater [^11^C]CURB λ*k*_3_ binding in a composite region sampling the medial prefrontal cortex, orbitofrontal cortex, anterior cingulate cortex, ventral striatum, substantia nigra, and remainder of midbrain (*r* = 0.60, *p* = 0.02).

**Fig. 3:**
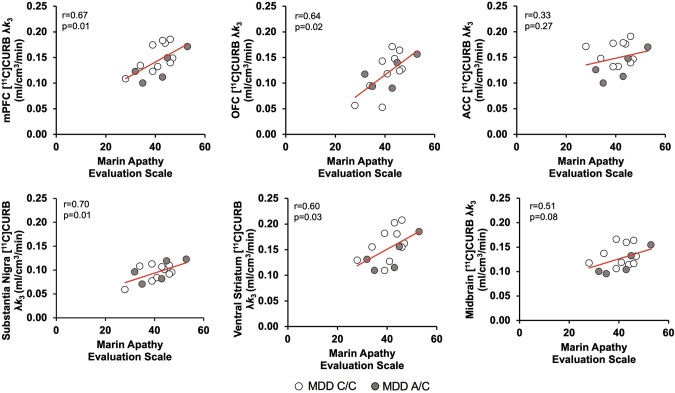
Positive correlations between Marin Apathy Evaluation Scale (MAES) scores and regional [^11^C]CURB λ*k*_3_ in the MDD group. MDD participants who had higher MAES scores also had greater [^11^C]CURB λ*k*_3_ binding in the medial prefrontal cortex (mPFC), orbitofrontal cortex (OFC), ventral striatum, and substantia nigra (*r* = 0.60 to 0.70, *p* = 0.01 to 0.03) but less so in the anterior cingulate cortex (ACC; *r* = 0.33, *p* = 0.27) and midbrain (*r* = 0.51, *p* = 0.08).

MAES scores in the MDD group were a significant covariate of [^11^C]CURB λ*k*_3_ binding in the linear mixed effects model that included repeated measures in brain regions related to apathy, sampling the mPFC, OFC, ACC, ventral striatum, substantia nigra, and remainder of midbrain (*F*_1,11_ = 6.81, *p* = 0.02; Table 2) which is consistent with the correlation between MAES scores and a composite region of [^11^C]CURB λ*k*_3_ binding derived from these individual regions (*r* = 0.60, *p* = 0.02; Fig. 2). Correlation coefficients between MAES scores and [^11^C]CURB λ*k*_3_ binding in individual regions were higher in mPFC, OFC, ventral striatum, and substantia nigra (*r* = 0.60 to 0.70, *p* = 0.01 to 0.03; Fig. 3, Table 2), but less so in ACC (*r* = 0.33, *p* = 0.27) and midbrain (*r* = 0.51, *p* = 0.08; Fig. 3, Table 2). To assess the specificity of the relationship between λ*k*_3_ and the ventral striatum, substantia nigra, OFC, and mPFC, an exploratory linear mixed effects model was completed evaluating the interaction between region and covariation of the MAES including these same regions as well as several regions not directly implicated in apathy, such as the occipital cortex and temporal cortex. The interaction between region and covariation of MAES was significant (F_6,24_ = 5.15, *p* = 0.002). Correlations with subscales of the MAES including cognitive (lack of interest), behavioral (lack of action) and emotional (lack of emotion) were explored and higher correlations were observed in the medial prefrontal cortex, the orbitofrontal cortex, ventral striatum and substantia nigra in relation to the cognitive subscale were noted (Table S3). The mean MAES was 41, and the majority of the MDD sample (*n* = 12) had a MAES total score above 34, indicative of substantial apathy uncommon in the midst of health. Total HDRS score was not a significant covariate of [^11^C]CURB λ*k*_3_ binding in the PFC, ACC, and hippocampus (*F*_1,11_ < 0.01, *p* = 0.97; Table 2). HAM-A score was also not a significant covariate of [^11^C]CURB λ*k*_3_ binding in brain regions related to anxiety in the MDD group, including the OFC, ACC, hippocampus, insula, and amygdala (*F*_1,11_ = 0.75, *p* = 0.49; Table 2).

**Table 2:** Relationship between [^11^C]CURB λ*k*_3_ and symptom measures in MDD group.

Depressive Severity						
Hamilton Depression Rating Scale^a^	All Prioritized Regions^b^	mPFC	dlPFC	OFC	ACC	HPC
	*F*_1,11_ < 0.01 *p* = 0.97	*r* = −0.10	*r* = −0.22	*r* = 0.10	*r* = 0.30	*r* = −0.18
		*p* = 0.75	*p* = 0.47	*p* = 0.74	*p* = 0.32	*p* = 0.56

Apathy							
Marin Apathy Evaluation Scale^a^	All Prioritized Regions^b^	mPFC	OFC	ACC	Substantia Nigra	Midbrain^c^	Ventral Striatum
	*F*_1,11_ = 6.81 *p* = 0.02*	*r* = 0.67	*r* = 0.64	*r* = 0.33	*r* = 0.70	*r* = 0.51	*r* = 0.60
		*p* = 0.01*	*p* = 0.02*	*p* = 0.27	*p* = 0.01*	*p* = 0.08	*p* = 0.03*

Anxiety						
Hamilton Anxiety Rating Scale^a^	All Prioritized Regions^b^	OFC	ACC	HPC	Amygdala	Insula
	*F*_1,11_ = 0.75 *p* = 0.49	*r* = −0.13	*r* = −0.15	*r* = −0.13	*r* = 0.02	*r* = −0.05
		*p* = 0.67	*p* = 0.63	*p* = 0.68	*p* = 0.94	*p* = 0.86

*mPFC* medial prefrontal cortex, *dlPFC* dorsolateral prefrontal cortex, *OFC* orbitofrontal cortex, *ACC* anterior cingulate cortex, *HPC* hippocampus.

^a^Partial Pearson Correlation Coefficient.

^b^Mixed effects model analysis.

^c^Midbrain ROI not including the substantia nigra.

*p < 0.05 (p-values uncorrected).

## Discussion

PET imaging of the ECS has been extensively reviewed and, to our knowledge, this is the first study to investigate brain FAAH in humans with MDD [48–50]. No significant differences in [^11^C]CURB λ*k*_3_ were observed between groups. This suggests that brain FAAH levels, as measured by [^11^C]CURB PET, do not differ between MDD and healthy controls. Greater apathy scores were associated with greater [^11^C]CURB binding in the mPFC, OFC, ACC, ventral striatum, and midbrain within the MDD group. These findings have important implications for FAAH in relation to apathy in MDD.

Contrary to our hypothesis, no significant differences in [^11^C]CURB λ*k*_3_ were found between MDD participants and healthy controls across all brain ROIs. A lack of difference between groups could indicate no alterations in brain FAAH in MDD, or alternatively, if there is a subgroup with elevated [^11^C]CURB λ*k*_3_ diluted by the heterogeneity of MDD, it is not prevalent enough to be detected in a group comparison. Were an elevation in FAAH present in the current study, it may have been expected that the presumed accompanying dysregulation of ECS could be reversed with FAAH inhibitors. Hence the negative findings of the present study could be considered consistent with the two negative clinical trials of FAAH inhibitors in MDEs [51, 52]. However, there are some limitations for this interpretation. One issue is that only one of the compounds, JNJ-42165279, has demonstrated high trough occupancy of approximately 80% during clinically relevant dosing in vivo in humans [53] whereas occupancy of SSR411298 is not known. In addition, there are limitations as to how the participant demographics of the clinical trials corresponded to the present study. The Phase II trial of SSR411298 sampled elderly patients with MDD [51] and, given the differences in the neurobiology and depressive symptom profiles between younger and older adults [54], the results of this clinical trial may not be generalizable. The other clinical trial using JNJ-42165279 in MDD with anxious distress [52] had no plan to selectively include patients with apathy, as the present study data was not available at the time of the study design, so the proportion of cases with apathy is not known.

The current study found a positive correlation between apathy and [^11^C]CURB λ*k*_3_ in the PFC and ventral striatum. It could be speculated that elevated FAAH in the PFC and basal ganglia is detrimental to the function of the prefrontal-subcortical circuit, which may manifest as a diminution of motivation and goal-oriented behavior, thus leading to apathy. For example, AEA, a key FAAH substrate, may influence dopamine releasing neurons projecting from the substantial nigra to the dorsal putamen and from ventral tegmental area to the nucleus accumbens through altering release of glutamate and GABA neurotransmitters at these sites [55, 56]. Under such circumstances it is possible that a FAAH inhibitor might be therapeutic. Some caution to this perspective should be given, however, since there was no group difference between MDD and controls in the current study. Similarly, while FAAH has been shown to be elevated in animal models of anxiety in previous studies [57], the lack of correlation between [^11^C]CURB λ*k*_3_ and anxiety in the MDD group may suggest that FAAH does not correspond to the anxiety associated with MDD in humans.

There are several limitations to the current study. First, due to the study’s cross-sectional design, we were unable to evaluate whether levels of brain FAAH change over time or under varying circumstances in our model. Second, [^11^C]CURB λ*k*_3_ is an index of FAAH levels but also includes a component of non-displaceable binding; thus, in theory, changes in non-displaceable binding could influence the binding measure. However, approximately 90% of λ*k*_3_ is directly attributable to specific binding [20], therefore it would take a massive effect of non-displaceable binding to substantially influence [^11^C]CURB λ*k*_3_. A third limitation is that we did not pursue measurement of anhedonia, nor measurement of plasma anandamide, which could have been assessed in relation to λ*k*_3_ and could be a direction for future study. Fourth, it is acknowledged that correlational analyses between indices of FAAH level and behavior may not capture complexities in the relationship between available FAAH levels and behavior, an issue exemplified in two relatively recent negative studies of FAAH inhibitors for PTSD [58, 59], despite beneficial effects on rodent fear extinction models. In addition, while several regions implicated in apathy circuitry had substantial correlations between λ*k*_3_ and the MAES, the ACC did not. This would not preclude a potentially causal role for FAAH level on apathy in MDD as it is possible that changes in FAAH in some regional components of apathy circuitry are sufficient to influence apathy, particularly when other components of illness are present, a direction which could be evaluated further in preclinical models in the future. Finally, severity of anxiety was only modestly elevated in this sample of MDD participants so it is possible that correlations between anxiety and [^11^C]CURB λ*k*_3_ could be present in a sample with greater anxiety.

The present study sought to investigate levels of brain FAAH in individuals with MDD compared to healthy controls using [^11^C]CURB PET imaging. We did not find any significant differences in [^11^C]CURB binding between the two groups in any of the brain regions examined, nor any relationship to anxiety. However, there was a significant positive association between [^11^C]CURB λ*k*_3_ and apathy scores on the MAES. These findings have important implications for understanding the role of the ECS in apathy and motivation-related behavior. Future work may consider investigations of ECS in relation to apathy and shifting some focus of clinical trials of FAAH inhibitors in humans towards apathy.

## Supplementary information

Supplemental Material

## Data Availability

All data corresponding to the graphs presented during this study are included in this published article as supplementary files.
