# Supplementary material for: Fatty acid amide hydrolase in major depressive episodes: A [11C]CURB positron emission tomography study
Source: Neuropsychopharmacology. 2025 Jun 23;50(10):1536–43. doi: 10.1038/s41386-025-02150-y (PMC12339711; doi:10.1038/s41386-025-02150-y)
Supplement: Supplementary file 1 — Supplemental Material [file 41386_2025_2150_MOESM1_ESM.pdf]

Fatty Acid Amide Hydrolase in Major Depressive Episodes:
A [11C]CURB Positron Emission Tomography Study
Supplemental Material

METHODS

Statistical Analysis

The original power calculation showed more than 80% power for an estimated difference of 13% in λk_3 between groups of 24 cases. However, the confidence interval estimate of the difference between groups in supplemental table 2 shows that a 13% elevation in λk_3 in MDE of major depressive disorder is outside the 95% confidence intervals for the primary regions in our present data so we felt this was an appropriate point to stop further recruitment. The Marin Apathy Evaluation Scale did not correlate with the items from the HDRS that are associated with apathy such as diminished work/interest, psychomotor retardation, and anergy, nor with the regional λk_3. Three subfactors of the MAES have been identified including cognitive (lack of interest), behavioral (lack of action) and emotional (lack of emotion). We quantitated these subscales and report their correlations in supplemental table 3 below.

RESULTS

Supplemental Table 1. [^{11}C]CURB radiotracer activity injected, specific activity at the time of injection, and total mass injected.

	MDD (n=15)	HC (n=15)	T-Value[1]	*P* value
**Activity Injected** (MBq)	369.6 ± 38.6	376.9 ± 16.6	*t* = -0.67	0.51
**Specific Activity at Time of Injection** (TBq/mmol)	74.5 ± 42.4	75.0 ± 45.4	*t* = -0.03	0.98
**Mass Injected** (nmol)	7.36 ± 6.39	8.92 ± 9.32	*t* = -0.53	0.60

[1]Independent Samples t-test.
Abbreviations: MDD, major depressive disorder; HC, healthy control.

Supplemental Table 2. [^{11}C]CURB λk_3 across all brain regions in MDD and healthy control groups.

ROI	[^{11}C]CURB λk_3, mean (SD) mL/cm^3/min		Between-Group Difference (95% CI)	Percent Difference	*P* Value
	MDD (n=15)	HC (n=15)			
**dlPFC**	0.13 (0.02)	0.13 (0.02)	0.00 (-0.02 to 0.02)	3.03%	0.70
**vlPFC**	0.13 (0.03)	0.12 (0.02)	0.01 (-0.01 to 0.02)	3.74%	0.61
**mPFC**	0.14 (0.03)	0.14 (0.02)	0.00 (-0.02 to 0.02)	1.43%	0.81
**OFC**	0.12 (0.04)	0.12 (0.03)	0.00 (-0.02 to 0.02)	1.72%	0.83
**Frontal Pole**	0.11 (0.03)	0.11 (0.02)	0.00 (-0.02 to 0.02)	0.92%	0.91
**ACC**	0.15 (0.03)	0.14 (0.02)	0.01 (-0.01 to 0.03)	4.88%	0.44
**Brodmann Area 25**	0.13 (0.04)	0.13 (0.03)	0.00 (-0.02 to 0.02)	0.80%	0.86
**Temporal Cortex**	0.14 (0.03)	0.14 (0.02)	0.01 (-0.01 to 0.02)	4.23%	0.52
**Occipital Cortex**	0.13 (0.03)	0.12 (0.02)	0.01 (-0.01 to 0.03)	7.47%	0.37
**Hippocampus**	0.16 (0.04)	0.15 (0.02)	0.01 (-0.01 to 0.03)	5.71%	0.31
**Amygdala**	0.16 (0.04)	0.16 (0.02)	0.00 (-0.02 to 0.02)	0.64%	0.93
**Insula**	0.15 (0.03)	0.15 (0.02)	0.00 (-0.01 to 0.02)	3.39%	0.58
**Ventral Striatum**	0.15 (0.03)	0.15 (0.03)	0.00 (-0.02 to 0.02)	1.43%	0.82
**Dorsal Caudate**	0.14 (0.03)	0.14 (0.02)	0.00 (-0.02 to 0.02)	2.18%	0.76
**Putamen**	0.16 (0.03)	0.16 (0.02)	0.00 (-0.02 to 0.02)	0.11%	0.99
**Thalamus**	0.16 (0.03)	0.16 (0.02)	0.00 (-0.02 to 0.02)	1.68%	0.77
**Midbrain**	0.13 (0.02)	0.13 (0.02)	0.01 (-0.01 to 0.03)	4.65%	0.89
**Substantia Nigra**	0.09 (0.02)	0.09 (0.01)	0.01 (-0.01 to 0.03)	8.03%	0.44
**Pons**	0.08 (0.02)	0.08 (0.02)	0.00 (-0.02 to 0.02)	0.52%	0.96
**Cerebellar Cortex**	0.16 (0.04)	0.14 (0.03)	0.01 (-0.01 to 0.03)	8.70%	0.14

Abbreviations: dlPFC, dorsolateral prefrontal cortex; vlPFC, ventrolateral prefrontal cortex; mPFC, medial prefrontal cortex; OFC, orbitofrontal cortex; ACC, anterior cingulate cortex; MDD, major depressive disorder; HC, healthy control.

Supplemental Table 3. Correlations between Marin Apathy Evaluation Scale (MAES) Subscale Scores and Total Scores with regional [^{11}C]CURB λk_3 in the MDD group.

	MAES Subscale Score[1]			MAES Total Score[1]
**ROI**	Cognitive	Behavioural	Emotional	
mPFC	r=0.62, p=0.01	r=0.19, p=0.49	r=0.34, p=0.21	r=0.67, p=0.01
OFC	r=0.68, p=0.01	r=0.27, p=0.34	r=0.46, p=0.08	r=0.64, p=0.02
Ventral Striatum	r=0.55, p=0.03	r=0.36, p=0.19	r=0.31, p=0.26	r=0.60, p=0.03
Substantia Nigra	r=0.53, p=0.04	r=0.02, p=0.93	r=0.29, p=0.29	r=0.70, p=0.01
ACC	r=0.30, p=0.28	r=0.25, p=0.38	r=0.04, p=0.89	r=0.33, p=0.27
Midbrain	r=0.43, p=0.11	r=0.24, p=0.39	r=0.23, p=0.41	r=0.51, p=0.08

[1] Pearson Correlation Coefficient.
[2]Midbrain ROI not including the Substantia Nigra.
